# Nerve damage induced skeletal muscle atrophy is associated with increased accumulation of intramuscular glucose and polyol pathway intermediates

**DOI:** 10.1038/s41598-020-58213-1

**Published:** 2020-02-05

**Authors:** Henning Tim Langer, Shoaib Afzal, Stefan Kempa, Simone Spuler

**Affiliations:** 1 Charité – Universitätsmedizin Berlin, corporate member of Freie Universität Berlin, Humboldt-Universität zu Berlin, and Berlin Institute of Health, Berlin, Germany; 2 Berlin-Brandenburg Center for Regenerative Therapies, Charité - Universitätsmedizin Berlin, Berlin, Germany; 3 Muscle Research Unit, Experimental and Clinical Research Center, a Joint Cooperation of Max Delbrück Center for Molecular Medicine and Charité - Universitätsmedizin Berlin, Berlin, Germany; 4 Berlin Institute of Health, Berlin, Germany; 5 Max Delbrück Center for Molecular Medicine in the Helmholtz Association, Berlin, Germany; 6 Department of Physiology and Membrane Biology, University of California Davis, Davis, CA USA

**Keywords:** Metabolomics, Neurophysiology

## Abstract

Perturbations in skeletal muscle metabolism have been reported for a variety of neuromuscular diseases. However, the role of metabolism after constriction injury to a nerve and the associated muscle atrophy is unclear. We have analyzed rat tibialis anterior (TA) four weeks after unilateral constriction injury to the sciatic nerve (DMG) and in the contralateral control leg (CTRL) (n = 7) to investigate changes of the metabolome, immunohistochemistry and protein levels. Untargeted metabolomics identified 79 polar metabolites, 27 of which were significantly altered in DMG compared to CTRL. Glucose concentrations were increased 2.6-fold in DMG, while glucose 6-phosphate (G6-P) was unchanged. Intermediates of the polyol pathway were increased in DMG, particularly fructose (1.7-fold). GLUT4 localization was scattered as opposed to clearly at the sarcolemma. Despite the altered localization, we found GLUT4 protein levels to be increased 7.8-fold while GLUT1 was decreased 1.7-fold in nerve damaged TA. PFK1 and GS levels were both decreased 2.1-fold, indicating an inability of glycolysis and glycogen synthesis to process glucose at sufficient rates. In conclusion, chronic nerve constriction causes increased GLUT4 levels in conjunction with decreased glycolytic activity and glycogen storage in skeletal muscle, resulting in accumulation of intramuscular glucose and polyol pathway intermediates.

## Introduction

Skeletal muscle atrophy is a pathological condition associated with many diseases. While protein metabolism is thought to be the main regulator of muscle size, many situations of atrophy and neuromuscular diseases are accompanied by changes to substrate metabolism as well. Critical illness myopathy (CIM) is a condition for which disturbances of glucose metabolism have been reported by our laboratory and others^1^. Specifically, we have found the primary glucose transporter GLUT4 to be insufficiently translocated in CIM patients, resulting in decreased glucose supply and reduced AMPK activity^2^. Besides CIM, a number of other neuromuscular disorders have been found to be show signs of changes to glucose metabolism in skeletal muscle such as amyotrophic lateral sclerosis (ALS), Charcot-Marie-Tooth neuropathy (CMT) or spinal muscular atrophy (SMA)^3–5^. For example, it has been found that concentrations of glucose as well as fructose are increased in skeletal muscle samples of ALS patients, often accompanied by an early onset of insulin resistance^6^. Early research in respect to nerve damage and glucose metabolism has reported that denervation is followed by insulin resistance, reduced glucose transport into the muscle, less glucose abundance and transiently decreased GLUT4 levels^7–9^. A more recent study in mice found that despite decreased GLUT4 mRNA abundance, long term denervation was associated with increased GLUT4 protein levels potentially regulated by increased Akt activity^10^. The same study found that glucose uptake in long term denervated muscle is increased compared to healthy controls^10^. However, data on changes to substrate metabolism after nerve damage are often limited to denervation via transection. By contrast, peripheral nerve damage in clinical practice often comprises a wide range of other injuries such as crush- or chronic constriction injuries^11^. These types of nerve injuries are clinically distinct from nerve transection and little is known on substrate metabolism in those conditions. We investigated changes to substrate metabolism in rat TA four weeks after constriction injury of the sciatic nerve as a follow-up to previous experiments on protein metabolism in the same animals^12^. We utilized a combination of untargeted metabolomics, immunohistochemistry and western blotting to examine alterations and potentially underlying mechanisms.

## Methods

### Ethical approval

All animal experiments were approved by the local authority (Landesamt für Gesundheit und Soziales, Berlin, Germany) under the reference G 0083/15 and performed at the animal care unit of the Max Delbrück Center for Molecular Medicine (MDC, Berlin). All experiments were carried out in accordance with local guidelines for good scientific practice and animal care.

### Animals

We have previously described the constriction injury model and skeletal muscle atrophy in the same animals that were investigated in this study^12^. Briefly, male Sprague-Dawley rats (Charles River, Germany) between 20–21 weeks of age were housed in individual cages. The animals were fed a chow diet suited towards weight maintenance rather than commonly occurring, age associated weight gain. Nerve damage and muscle atrophy were induced via chronic constriction injury by implanting an electrode cuff around the sciatic nerve slightly above the point of trifurcation.

### Free metabolite extraction

The free metabolite pool of muscle samples was extracted as described previously with small modifications^13^. Frozen tissue of rat TA (n = 7) was weighed and 20 mg of tissue per sample was solubilized in methanol-chloroform-water (MCW; 5:2:1) (Sigma-Aldrich, Germany), containing 2 µg/ml cinnamic acid (Sigma-Aldrich, Germany) as internal standard. After addition of MCW, all samples were shaken twice at 6000RPM for 1 min using a Precellys 24 (Bertin Technologies, France) to homogenize them. This step was repeated after 10 µl ddH2O * mg-1 of tissue was added to the muscle samples. For phase separation of polar and lipid intermediates, all samples were spun for 10 min at 20,000 g and 4 °C. The supernatant containing the polar phase was then collected and dried for 3 h in a SpeedVac (Thermo Scientific, Germany). For derivatization, the dried samples were dissolved in 20 µl methoxyamine hydrochloride solution (Sigma-Aldrich, Germany) (40 mg/ml in pyridine [Roth, Germany]) and incubated for 90 min at 30 °C in a Thermomixer (Eppendorf, Germany). Subsequently, 80 µl of N-methyl-N- (trimethylsilyl)trifluoroacetamide (MSTFA) (Machery-Nagel, Germany) containing a retention index standard was added to the samples and incubated at 37° for 45 min. The supernatant was then collected and transferred into appropriate vials for GC-MS measurement.

### Untargeted metabolomics

GC-TOF-MS measurements were performed as described previously^14^. Briefly, a gas chromatograph coupled to a time of flight mass spectrometer (Pegasus III- TOF-MS-System [LECO, USA]) was used for analysis. The samples were prepared as described in the section above and injected into the GC-TOF-MS in a split mode using a temperature-controlled injector (Gerstel, Germany) with a baffled glass liner (Gerstel, Germany). For free metabolite concentrations we used a previously established split of 1:5 and samples were injected once. After initially starting the injection at 80 °C for 30 s, the temperature was gradually increased up to 300 °C for 2 min. Gas chromatographic separation was performed on an Agilent 6890 N (Agilent, USA), equipped with a VF 5 ms column (Varian, USA). Helium was used as carrier gas at 1.2 ml/min flow rate. Gas chromatography was performed with a temperature starting at 70 °C for 2 min, gradually increasing up to 350 °C for 2 min. At a detector voltage of 1750 V, spectra were recorded in a mass range of 60 to 600 u with 20 spectra/s. The data were pre-processed with ChromaTOF (LECO, USA) including resampling, baseline subtraction and peak detection. Subsequently the data were read into our house intern software MAUI-VIA for annotation and quantification of individual metabolites, more elaborately described elsewhere^15^.

### Immunohistochemistry

Cryosections for immunohistochemistry have been prepared as described previously^12^. Sectioned samples were left 1 h at room temperature to dry, before being fixated in formaldehyde for 5 min. Sections were blocked in PBS (3% BSA) for 30 min, washed and subsequently incubated with GLUT4 (1:1000 in PBS (1% BSA), provided by A. Schürmann, DIfE Potsdam) for 1 h at room temperature. After washing, sections were incubated with a biotinylated secondary antibody (1:200 in PBS; Dianova, Germany) for 30 min, washed and incubated with Streptavidin-Cy3 (1:200 in PBS; Dianova, Germany) for 30 min. Subsequently the sections were washed and incubated with Hoechst (1:1000 in PBS; Thermo Fisher, Germany) for 5 min to stain for nuclear positioning, washed and mounted on slides using Aqua Mount (Polysciences, Germany).

### Western blotting

Rat TA (n = 5) was immersed in 150 mM NaCl, 0.5% Triton X-100, 50 mM Tris, 50 mM NaF, 1 mM Na3VO4, 1 mM PMSF, 0.5% Na-Deoxycholate, 1 mM EDTA, and 1x cOmplete protease inhibitor complex (Roche, Germany) and homogenized with a pestil. After lysing the samples they were centrifuged for 15 min (13,200 rpm) and the supernatant collected. Semi dry transfer was conducted as described previously^12^. The following antibodies were used: Dysferlin HAMLET (Novocastra, Germany), Cyclophillin A (#ab41684) (Abcam, UK), Akt (#9272), pAkt (#9271), AMPK (#2603), pAMPK (#2535) (Cell Signaling, USA). The secondary antibodies were applied accordingly and the membrane imaged via infrared imaging (Odyssey, LI-COR Biosciences, USA). We and others have previously found that normalizing protein levels to housekeeping proteins is less reliable than normalizing to total protein content^12,16,17^. We therefore normalized all levels of our proteins to total protein content of the gel stained with Coomassie Blue. During revisions, the same set muscle samples were freshly prepared according to a similar protocol as above and the sample size increased (n = 6)^18^. The following antibodies were used: IRS1 (#2382), pan NOS (#2977), GSK3 α/β (#9331), GS (#3893), Annexin A2 (#8235), LC3B (#2775) (Cell Signaling, USA), GLUT1 (#7903), GLUT4 (#53566), PFK1 (#31712), Muscle LIM (CRP3) (#166930), eNOS/NOS III (#610296) (BD Biosciences, USA) and total OXPHOS (#MS604–300) (Abcam, UK). All band intensities were normalized to total protein content of the gel as assessed via stain-free technology (Criterion TGX Stain-Free Gels, Bio-Rad, USA).

### Statistics

Groups were compared with an unpaired t test and results with a p value < 0.05 deemed statistically significant. Effect size was calculated according to Cohen: *d* = (M_1_ − M_2_)/SD_pooled_, where *d* is the effect size, M_1_ is the control leg and M_2_ the damaged leg and SD_pooled_ the combined standard deviation of all values from both groups for each metabolite. Data are displayed as mean and standard deviation (SD).

## Results

### Altered muscle metabolism in nerve damaged TA

**Figure 1:**
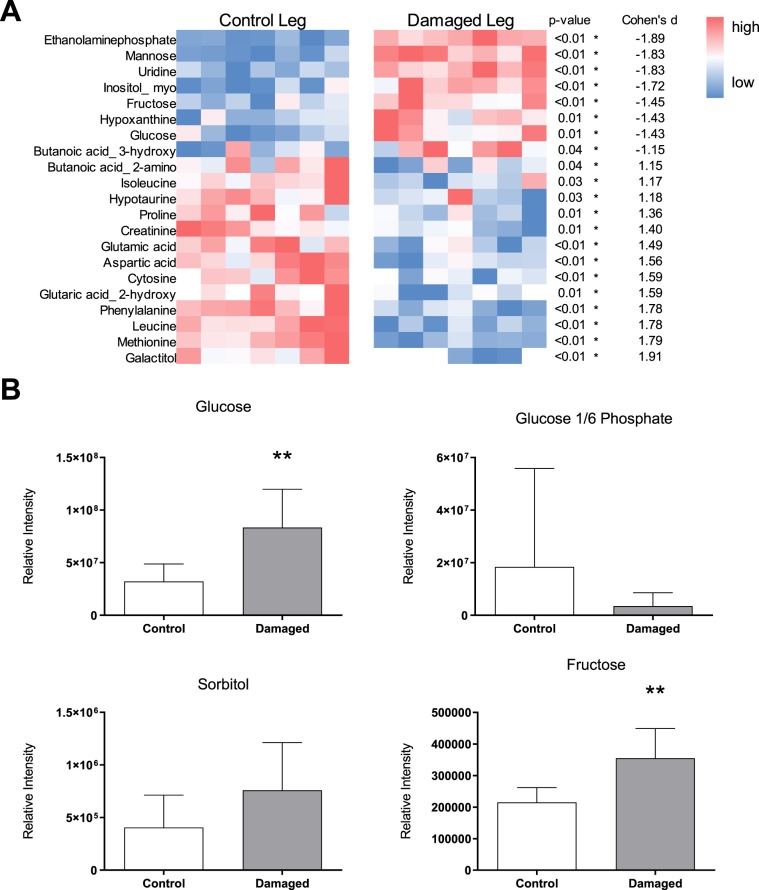
Untargeted metabolomics of rat TA four weeks after constriction injury to the sciatic nerve. (**A**) After annotation, 79 polar metabolites could be identified, 27 of which were significantly altered in nerve damaged TA (DMG) compared to the contralateral control leg of the same animals (CTRL). The heatmap summarizes all metabolites (only main products) which are significantly different between CTRL and DMG together with the corresponding effect size (d). Metabolites are sorted from low to high d. **(B)** Glucose was increased 2.6-fold in DMG compared to CTRL (p < 0.007). (**C**) G6-P was decreased 1.9-fold, without reaching statistical significance due to high variability. (**D**) Sorbitol was increased 1.9-fold, but could not be analyzed statistically as the concentrations in 5 out of 7 animals in the control leg were below the threshold of detection. (**E**) Fructose was increased 1.7-fold in DMG compared to control (p < 0.005). Groups were compared via unpaired t-test, *indicates p < 0.05, **indicates p < 0.01.

Rat TA (n = 7) was phase-separated and the polar phase, containing free intramuscular metabolites of glycolysis and amino acid metabolism, was subjected to untargeted metabolomics via GC-TOF-MS. We could identify 79 metabolites out of which 27 were significantly altered in nerve damaged TA (Fig. 1A; Supplement). Specifically, glucose metabolism appeared to be affected: glucose concentrations were increased 2.6-fold in the nerve damaged TA (Fig. 1B). Under physiological circumstances, glucose is phosphorylated to G6-P by hexokinase upon entering the cell. Therefore, increased concentrations of glucose would be expected to be accompanied by a concomitant increase in G6-P to feed glucose into glycolysis. However, G6-P appeared to be unchanged or slightly decreased rather than increased in damaged TA (Fig. 1B; d = 0.6). A substrate pathway which is upregulated in case of deranged or saturated glycolysis is the polyol pathway. Increased activity of this pathway should result in increased abundance of pathway intermediates and/or end products. Indeed we found that among those 27 differentially regulated metabolites in the nerve damaged TA, the polyol pathway end product fructose was upregulated 1.7-fold compared to the control leg (Fig. 1B). The intermediate sorbitol appeared increased in the damaged TA as well, but did not reach statistical significance (Fig. 1B). However, sorbitol could only be found and annotated in two out of seven animals in the control leg, indicating that concentrations in healthy muscle are below or only slightly above our detection threshold (Fig. 1B). In further support of the hypothesis that glucose metabolism is affected and other substrate pathways upregulated as a compensatory mechanism, we screened the differentially regulated metabolites for ketone bodies. We found butanoic acid 3-hydroxy to be upregulated 1.8-fold in nerve damaged TA (Supplement S1).

### Scattered localization of GLUT4 in nerve damaged TA

**Figure 2:**
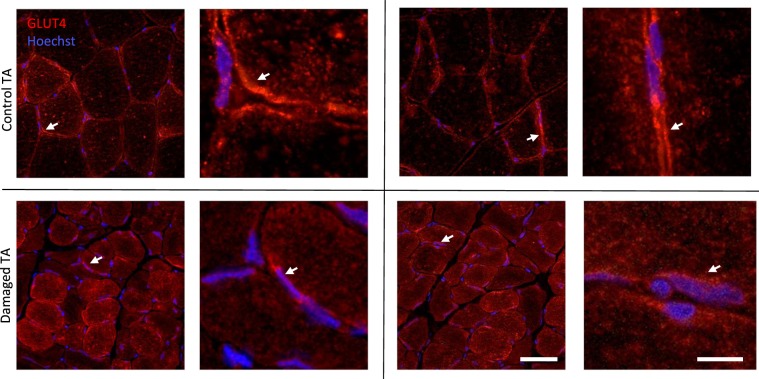
Altered GLUT4 localization in nerve damaged muscle. Nerve damage affected TA and controlateral control TA were sectioned and processed for immunohistochemical analysis of GLUT4. Stainings indicate that GLUT4 is more homogeneously located around the sarcolemma in healthy control muscle (white arrows), but scattered and aggregated in nerve damaged muscle with occasional accumulation in the perinuclear space (white arrows). Scale bars: 50 µm (left) and 10 µm (right).

We next investigated localization of GLUT4 by immunohistochemistry on nerve damaged rat muscle and contralateral control sections (Fig. 2). We found GLUT4 primarily located in the perinuclear space and unevenly distributed across damaged TA sections while in control sections GLUT4 was located to the sarcolemma (Fig. 2).

### Elevated levels of proteins associated with membrane damage, cytoskeletal integrity and oxidative activity

**Figure 3:**
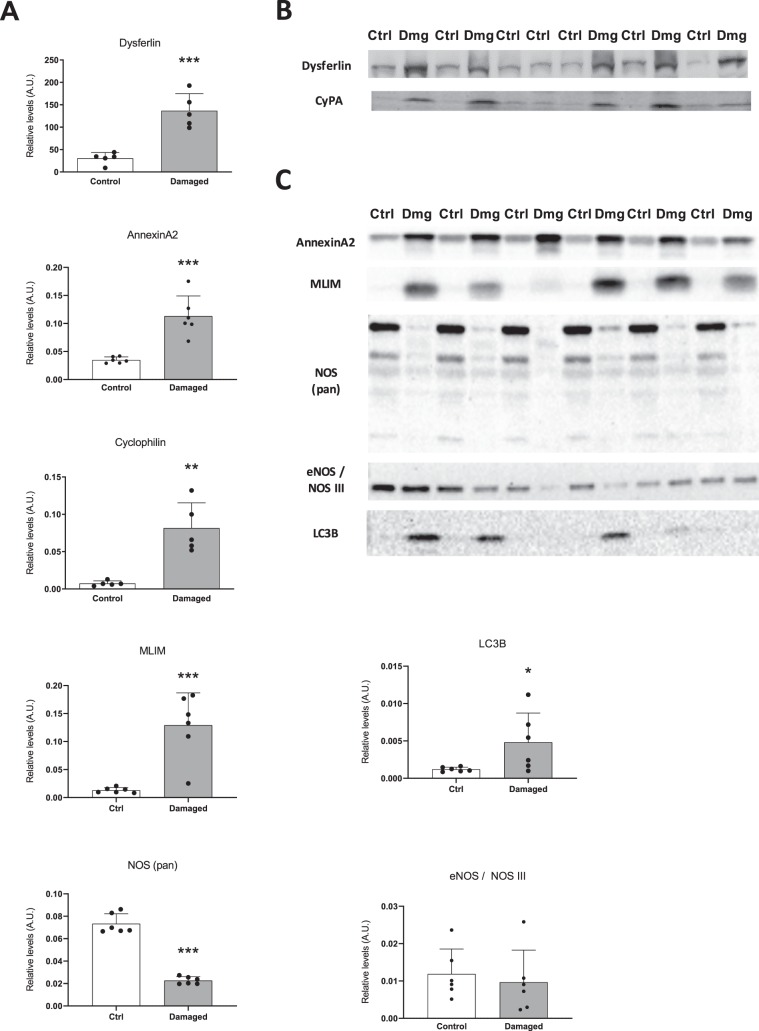
Protein levels associated with membrane damage, cytoskeletal integrity, inflammation and autophagy are increased in nerve damaged TA, while oxidative activity appears to be decreased. Rat TA was subjected to western blot anaylsis of proteins associated with muscle damage. (**A**) Dysferlin, AnnexinA2, Cyclophilin, MLIM and LC3B are significantly upregulated in nerve damaged TA, while NOS (pan) levels are substantially decreased with nerve damage. (**B**) Bands corresponding to the western blot quantification in (**A**) (n = 5). The cropped pictures displayed above show the entire width of the membrane and all samples included for several different proteins. In order to allow for probing of multiple proteins with distinct molecular weight simultaneously, the membranes where cut horizontally at the appropriate heights. All band intensities were normalized to total protein content of the gel as assessed via coomassie blue staining. (**C**) Bands corresponding to the western blot quantification in (**A**) (n = 6). These blots were added during a later stage of the manuscript. All band intensities were normalized to total protein content of the gel as assessed via stain-free technology. *p < 0.05, **p < 0.01, ***p < 0.001.

To investigate the underlying molecular changes of the observed structural damage to the muscle we performed western blot analysis. We found dysferlin, a big structural protein and a mediator of membrane repair, to be upregulated 4.5-fold in damaged TA (Fig. 3A,B). Annexin A2 is a protein involved in linkage of membrane proteins to the actin complex and closely associated with Dysferlin^19^. Annexin A2 was increased 3.3-fold in damaged TA compared to healthy controls (Fig. 3A,C). Cyclophilin A (CyPA) is a damage associated protein which is thought to contribute to inflammation and cell death and was found to be upregulated 11-fold in nerve damaged TA compared to the contralateral control (Fig. 3A,B). Muscle LIM (CRP3) has been shown to be involved in cytoskeletal integrity of the contractile apparatus by organizing and connecting myofibrils and interacting with proteins in the z-disc of sarcomeres, as well as a potential role in autophagy^20,21^. Muscle LIM was increased 9.8-fold in nerve damaged TA (Fig. 3A,C). In line with the potential role of Muscle LIM in autophagy, we found LC3B levels to be 4-fold increased with nerve damage (Fig. 3A,C). Interestingly, NOS (pan) levels were reduced 3.2-fold with nerve damage, indicating decreased oxidative activity (Fig. 3A,C). This is in contrast to the metabolomics data which indicated upregulated oxidative activity through increased polyol pathway utilization. All bands on the membrane (Fig. 3B,C) were normalized to total protein content per corresponding lane on the gel (Supplement S2 and S3) as described previously^12,16–18^.

### Increased glucose concentrations are accompanied by increased GLUT4 levels, but decreased glycolysis and glycogen synthesis

**Figure 4:**
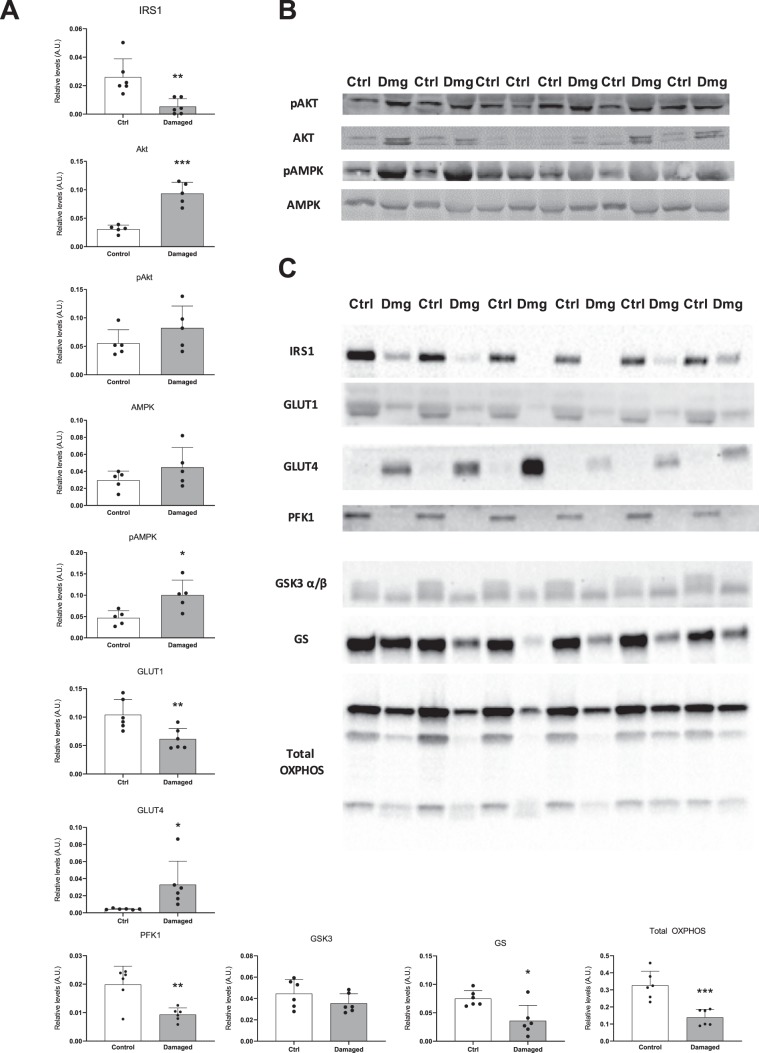
Altered protein levels associated with glucose metabolism and mitochondrial activity in nerve damaged TA. Rat TA was subjected to western blot anaylsis of proteins associated with glucose metabolism and oxidative metabolism. (**A**) IRS1 is downregulated, while total AKT is increased. Insulin independent GLUT4 signaling via pAMPK is slightly increased as well. Glucose transporter GLUT4 is increased, while GLUT1 levels are decreased in nerve damage. The rate limiting proteins of glycolysis (PFK1) and glycogen synthesis (GS) are decreased with nerve damaged TA. Additionally, total oxidative phosphorylation of mitochondrial proteins is decreased in nerve damage. (**B**) Bands corresponding to the western blot quantification in (**A**) (n = 5). All band intensities were normalized to total protein content of the gel. (**C**) Bands corresponding to the western blot quantification in (**A**) (n = 6). These blots were added at a later stage of the manuscript. All band intensities were normalized to total protein content of the gel. *p < 0.05, **p < 0.01, ***p < 0.001.

Nerve damage caused a 4.8-fold decrease in IRS1 signaling (Fig. 4A,C). Interestingly, despite this decrease, total- and pAkt levels were found to be increased 3-fold and 1.5-fold respectively (Fig. 4A,B). The scattered localization of GLUT4 found in Fig. 2 does not seem to impact GLUT4 protein levels, as GLUT4 was increased 7.8-fold in nerve damaged TA while GLUT1 was decreased 1.7-fold, indicating the primary mode of glucose transport is still through GLUT4 (Fig. 4A,C). Downstream of GLUT4, however, we found rate limiting proteins of glycolysis and glycogen synthesis to be substantially decreased in nerve damaged muscle. PFK1, phosphorylating fructose 6-phosphate to fructose 1/6 bisphosphate during glycolysis, was decreased 2.1-fold in nerve damaged TA (Fig. 4A,C). This decrease in PFK1 was accompanied by an equally great 2.1-fold decrease in GS, indicating that the increase in glucose concentrations found in the metabolomics data (Fig. 1B) is neither resulting in increased glycolysis nor glycogen storage. Finally, oxidative metabolism as represented by total OXPHOS levels was decreased 2.4-fold, pointing to the fact that besides glycolysis and glycogen synthesis, mitochondrial activity in skeletal muscle appears to be negatively affected by chronic nerve constriction as well.

## Discussion

The main purpose of this study was to investigate the changes occurring to skeletal muscle metabolism with chronic nerve constriction injury and relate them back to findings in nerve transection and other neuromuscular diseases. We found that chronic constriction injury of the sciatic nerve in rats results in increased intramuscular glucose and polyol pathway intermediate concentrations. The transport of glucose appeared to be primarily mediated through GLUT4, even though localization around the sarcolemma was scattered. The increase in glucose and GLUT4 was accompanied by a decrease in PFK1 and GS, indicating that glycolysis and glycogen synthesis are unable to process glucose at sufficient rates.

That neuromuscular diseases can affect skeletal muscle metabolism is long known^4^. For example, ALS has been shown to be accompanied by insulin resistance and accumulation of glucose and fructose in muscle samples of patients^3^. Interestingly, we have found IRS1 levels in our rats to be substantially decreased with nerve damage, while glucose and fructose concentrations were increased. Since ALS is defined as the progressive deterioration and eventual death of motor neurons, it is not inconceivable that the metabolic phenotype observed in skeletal muscle of these patients could be a function of nerve damage and shares certain characteristics with a peripheral nerve disorder such as the constriction injury in our model.

When compared to nerve transection previous research suggests a significant overlap with our data. Early research found that nerve transection induced insulin resistance and was accompanied by a decreased ability of skeletal muscle to take up glucose^8^. While this was further reflected by decreased glucose concentrations and GLUT4 levels^7^, more recent studies have indicated that this effect might be transient^10^. Callahan *et al*. have shown that transection of the sciatic nerve and the associated muscle loss in mice are followed by a decrease in GLUT4 mRNA expression and an inability of insulin to stimulate glucose uptake 3 days after surgery^10^. However, at 28 days post surgery, while continuing to lose muscle mass and cross sectional area, the mice still had decreased GLUT4 mRNA expression, but started to show increased GLUT4 protein levels and an improved ability to take up glucose with and without insulin^10^. This effect corresponds well with the timeline of our study and appears to become exacerbated over time, despite continuous muscle loss, as the group differences in the Callahan study further increased at 56 days post surgery. While we have not tested insulin sensitivity directly, the decreased IRS1 levels we found with nerve constriction injury in our rats might indicate slight differences to the aforementioned nerve transection study in mice. In spite of this, the authors have found the effect in increased glucose uptake and GLUT4 to coincide with increased total Akt and pAkt levels, which is in line with our findings as well (Fig. 4A,B).

It is important to point out that for GLUT4’s ability to modulate glucose uptake, localization at the sarcolemma had been shown to be of greater importance than protein abundance^22^. Since we have seen abundant, but scattered localization of GLUT4 with constriction injury to the nerve (Fig. 2), we were initially skeptical towards the ability of GLUT4 to function as an efficient glucose transporter in these animals. However, several lines of evidence point to a sufficient and likely even increased uptake of glucose via GLUT4 in our model. The first important indicator that GLUT4 is still the primary route of glucose transport is the decrease observed in GLUT1 levels in nerve damaged TA (Fig. 4A,C). These are the two most prevalent glucose transporter in skeletal muscle and the 1.7-fold decrease in GLUT1 coincides with a 7.8-fold increase in GLUT4. Furthermore, two proteins downstream of glucose transport, specifically PFK1 and GS indicate a decrease in glycolysis and glycogen synthesis. This is in line with the metabolomics data, where we measured increased intramuscular glucose concentrations that were not accompanied by an increase in G6-P (Fig. 1A). Lastly, the evidence provided by a similar nerve damage model found an increase in GLUT4 that was associated with improved glucose uptake^10^. Therefore, glucose transport into muscle affected by constriction injury to the nerve appears to be primarily governed through GLUT4, despite its scattered localization.

Another disease sharing certain characteristics of a metabolic phenotype with our study, despite not being a neuromuscular disorder per se, is diabetes mellitus. Even though diabetes is defined by whole body insulin resistance, skeletal muscle as the greatest glycogen depot of the human body has still a particularly great role in the development and progression of the disease. Several studies in diabetic mice and -men have shown increased polyol pathway activity in different tissues^23^. This increased activity of polyol pathway intermediates such as sorbitol and fructose were suggested to contribute to oxidative damage and eventually polyneuropathy in animals and patients^24–26^. In our study, we found decreased IRS1 signaling and increased concentrations of polyol pathway intermediates sorbitol and fructose with nerve damage (Fig. 1A). Therefore we were intrigued whether this upregulated activity of the polyol pathway was associated with increased oxidative damage, potentially explaining part of the muscle loss we have seen in our animals previously^12^. Surprisingly, we found total NOS activity to be reduced >3-fold in nerve damaged TA (Fig. 3A,C). While we cannot conclusively discard the possibility that increased polyol pathway activity contributes to oxidative damage and muscle atrophy in our model, the decrease in total NOS makes it appear less likely and points towards a more physiological role of the polyol pathway.

Indeed, in another neurological disease associated with motor neuron damage, muscle atrophy and a metabolic phenotype, Charcot-Marie-Tooth disease (CMT), the polyol pathway intermediate sorbitol is currently tested in a phase III clinical trial as part of a combination of treatments for CMT type 1 A (NCT03023540). Previous data in animals and a phase II trial suggest a moderate effect on myelination and muscular performance, particularly when sorbitol is used in conjunction with the other two treatment components (baclofen, naltrexone)^27–29^. While it seems unlikely that sorbitol alone would exhibit a substantial effect on disease progression and muscle loss for any neuromuscular disease, it does indicate that there could be a physiological and potentially even mildly beneficial role for sorbitol as a substrate in those conditions. Further supporting this notion, co-ingestion of sorbitol with glucose has recently shown improved blood glucose levels and muscle glucose uptake *ex vivo* in healthy and diabetic rats compared to glucose ingestion alone^30^. If these findings turn out to be biologically relevant in humans, then alternative fuels could be an exciting avenue to pursue as an adjunct therapeutic approach or at least for refined nutritional recommendations toward patients. Relating to the idea of alternative fuels for degenerating muscle, we have also found beta-hydroxybutyrate to be elevated in nerve damaged TA (Supplemental Fig. S1). Thus, the polyol pathway might not be the only interesting candidate to investigate.

While the underlying pathology causing the metabolic phenotype in diseases such as ALS, CMT, nerve transection and nerve constriction could intuitively be attributed to gradual denervation, this common denominator is not present in other neuromuscular diseases that still share some of the same metabolic characteristics. For example, previous research has shown that dermatomyositis (DM) and its disease progression are at least partially associated with increased dysferlin levels as well as increased polyol pathway activity^31^. Dysferlin is a large membrane protein and important for the integrity of the sarcolemma and injury repair^32^. We have found dysferlin and several other proteins with a role in muscle damage to be elevated in our model (Fig. 3A–C). Despite this correlation between increased dysferlin levels and polyol pathway activity, data obtained from dysferlin deficient mice and primary myoblasts from dysferlinopathy patients indicate certain similarities with the metabolic phenotype seen in our nerve damage model^33^. Interestingly, a similar pattern can be observed for Duchenne muscular dystrophy (DMD). While there are reports of impaired glucose uptake and increased insulin resistance in DMD patients^34^, a recent study in a golden retriever model for Duchenne (GRMD) has found decreased GLUT4 mRNA expression, but increased GLUT4 protein levels and glucose flux into GRMD skeletal muscle^35^. Therefore, skeletal muscle metabolism is evidently highly complex and whether the metabolic phenotype observed in our and other models of nerve damage is primarily a function of neurodegeneration or rather a symptom of muscle that undergoes remodeling and atrophy is unclear. Indeed, we have shown that the metabolic phenotype we report here is also associated with increased myofibrillar protein synthesis and histopathological markers of muscle remodeling in the same animals^12^.

To conclude, peripheral nerve disorders, motor neuron diseases, inheritable neurological diseases and neuromuscular diseases share certain characteristics of deranged glucose metabolism. These include altered insulin sensitivity, GLUT4 expression, -translocation and protein levels, as well as increased glucose uptake into muscle. We have shown that chronic constriction injury to the sciatic nerve causes increased intramuscular glucose- and polyol pathway intermediate concentrations. While traditionally thought to be undesirable, the decrease of NOS levels in our model and the results of other recent studies suggest that there might be a physiological role for the polyol pathway in damaged muscle. The observed increase in glucose is not accompanied by increased G6-P, but by elevated levels of GLUT4 and a concomitant decrease of the rate limiting enzymes of glycolysis (PFK1) and glycogen synthesis (GS). Thus, glucose and fructose accumulation in nerve damaged muscle appear to be a combination of increased glucose uptake and decreased glycolytic activity. Future research needs to decipher whether these metabolic changes are caused by neurodegeneration or whether they are a common feature of muscle atrophy and remodeling.

## Supplementary information

Supplementary Dataset 1.

## References

[1] Ochala J (2011). Preferential skeletal muscle myosin loss in response to mechanical silencing in a novel rat intensive care unit model: underlying mechanisms. The Journal of physiology.

[2] Weber-Carstens S (2013). Critical illness myopathy and GLUT4: significance of insulin and muscle contraction. American journal of respiratory and critical care medicine.

[3] Mueller PS, Quick DT (1970). Studies of glucose, insulin, and lipid metabolism in amyotrophic lateral sclerosis and other neuromuscular disorders. The Journal of laboratory and clinical medicine.

[4] Collis WJ, Engel WK (1968). Glucose metabolism in five neuromuscular disorders. Neurology.

[5] Pich S (2005). The Charcot–Marie–Tooth type 2A gene product, Mfn2, up-regulates fuel oxidation through expression of OXPHOS system. Human molecular genetics.

[6] Poulton K, Rossi M (1993). Peripheral nerve protein glycation and muscle fructolysis: evidence of abnormal carbohydrate metabolism in ALS. Functional neurology.

[7] Block NE, Menick DR, Robinson KA, Buse MG (1991). Effect of denervation on the expression of two glucose transporter isoforms in rat hindlimb muscle. The Journal of clinical investigation.

[8] Davis TA, Karl IE (1988). Resistance of protein and glucose metabolism to insulin in denervated rat muscle. The Biochemical journal.

[9] Buse MG, Buse J (1959). Glucose uptake and response to insulin of the isolated rat diaphragm: the effect of denervation. Diabetes.

[10] Callahan Zachary J., Oxendine Michael, Wheatley Joshua L., Menke Chelsea, Cassell Emily A., Bartos Amanda, Geiger Paige C., Schaeffer Paul J. (2015). Compensatory responses of the insulin signaling pathway restore muscle glucose uptake following long-term denervation. Physiological Reports.

[11] Dyck, P. *Peripheral neuropathy*. (Elsevier Inc., 2005).

[12] Langer HT (2018). Muscle atrophy due to nerve damage is accompanied by elevated myofibrillar protein synthesis rates. Frontiers in Physiology.

[13] Pietzke M, Zasada C, Mudrich S, Kempa S (2014). Decoding the dynamics of cellular metabolism and the action of 3-bromopyruvate and 2-deoxyglucose using pulsed stable isotope-resolved metabolomics. Cancer & metabolism.

[14] Park TJ (2017). Fructose-driven glycolysis supports anoxia resistance in the naked mole-rat. Science.

[15] Kuich PH, Hoffmann N, Kempa S (2014). Maui-VIA: A User-Friendly Software for Visual Identification, Alignment, Correction, and Quantification of Gas Chromatography-Mass Spectrometry Data. Frontiers in bioengineering and biotechnology.

[16] Gilda JE, Gomes AV (2013). Stain-Free total protein staining is a superior loading control to β-actin for Western blots. Analytical biochemistry.

[17] Aldridge GM, Podrebarac DM, Greenough WT, Weiler IJ (2008). The use of total protein stains as loading controls: an alternative to high-abundance single-protein controls in semi-quantitative immunoblotting. Journal of neuroscience methods.

[18] West DW (2016). Acute resistance exercise activates rapamycin‐sensitive and‐insensitive mechanisms that control translational activity and capacity in skeletal muscle. The Journal of physiology.

[19] Defour A (2017). Annexin A2 links poor myofiber repair with inflammation and adipogenic replacement of the injured muscle. Human molecular genetics.

[20] Vafiadaki E, Arvanitis DA, Sanoudou D, Muscle LIM (2015). Protein: Master regulator of cardiac and skeletal muscle functions. Gene.

[21] Rashid MM (2015). Muscle LIM protein/CSRP3: a mechanosensor with a role in autophagy. Cell death discovery.

[22] Gibbs EM (1995). Glycemic improvement in diabetic db/db mice by overexpression of the human insulin-regulatable glucose transporter (GLUT4). The Journal of clinical investigation.

[23] Tang WH, Martin KA, Hwa J (2012). Aldose reductase, oxidative stress, and diabetic mellitus. Frontiers in pharmacology.

[24] Amano S (2002). Sorbitol dehydrogenase overexpression potentiates glucose toxicity to cultured retinal pericytes. Biochemical and biophysical research communications.

[25] Obrosova IG (2005). Increased sorbitol pathway activity generates oxidative stress in tissue sites for diabetic complications. Antioxidants & redox signaling.

[26] Aquilano K (2007). Reactive oxygen and nitrogen species are involved in sorbitol-induced apoptosis of human erithroleukaemia cells K562. Free radical research.

[27] Prukop T (2019). Early short-term PXT3003 combinational therapy delays disease onset in a transgenic rat model of Charcot-Marie-Tooth disease 1A (CMT1A). PloS one.

[28] Chumakov I (2014). Polytherapy with a combination of three repurposed drugs (PXT3003) down-regulates Pmp22 over-expression and improves myelination, axonal and functional parameters in models of CMT1A neuropathy. Orphanet J Rare Dis.

[29] Attarian S (2014). An exploratory randomised double-blind and placebo-controlled phase 2 study of a combination of baclofen, naltrexone and sorbitol (PXT3003) in patients with Charcot-Marie-Tooth disease type 1A. Orphanet journal of rare diseases.

[30] Chukwuma CI, Islam MS (2017). Sorbitol increases muscle glucose uptake *ex vivo* and inhibits intestinal glucose absorption *ex vivo* and in normal and type 2 diabetic rats. Applied physiology, nutrition, and metabolism = Physiologie appliquee, nutrition et metabolisme.

[31] Xiao Yizhi, Zhu Honglin, Li Liya, Gao Siming, Liu Di, Dai Bingying, Li Qiuxiang, Duan Huiqian, Yang Huan, Li Quanzhen, Zhang Huali, Luo Hui, Zuo Xiaoxia (2019). Global analysis of protein expression in muscle tissues of dermatomyositis/polymyosisits patients demonstrated an association between dysferlin and human leucocyte antigen A. Rheumatology.

[32] Bansal D (2003). Defective membrane repair in dysferlin-deficient muscular dystrophy. Nature.

[33] Keller, S. *GC/MS-and LC/MS-based metabolic and proteomic analysis of dysferlin-deficient muscle from patients and animal models*, Freie Universität Berlin (2014).

[34] Freidenberg GR, Olefsky JM (1985). Dissociation of insulin resistance and decreased insulin receptor binding in Duchenne muscular dystrophy. The Journal of clinical endocrinology and metabolism.

[35] Schneider SM (2018). Glucose Metabolism as a Pre-clinical Biomarker for the Golden Retriever Model of Duchenne Muscular Dystrophy. Molecular imaging and biology.

