# Supplementary material for: Nerve damage induced skeletal muscle atrophy is associated with increased accumulation of intramuscular glucose and polyol pathway intermediates
Source: Sci Rep. 2020 Feb 5;10:1908. doi: 10.1038/s41598-020-58213-1 (PMC7002415; doi:10.1038/s41598-020-58213-1)
Supplement: Supplementary file 1 — Supplementary Dataset 1. [file 41598_2020_58213_MOESM1_ESM.docx]

**Supplemental File**

**Nerve damage induced skeletal muscle atrophy is associated with increased accumulation of intramuscular glucose and polyol pathway intermediates**

*Langer Henning Tim^1,2,3*^, Afzal Shoaib^1^, Kempa Stefan^4,5^, Spuler Simone,^1,3,4,5^.*

*^1^ Muscle Research Unit, Experimental and Clinical Research Center, a Joint Cooperation of Max Delbrück Center for Molecular Medicine and Charité - Universitätsmedizin Berlin, Berlin, Germany.*

*^2^Berlin-Brandenburg Center for Regenerative Therapies, Charité - Universitätsmedizin Berlin, Berlin, Germany.*

*^3^Charité - Universitätsmedizin Berlin, Berlin, Germany.*

*^4^Berlin Institute of Health, Berlin, Germany.*

*^5^Max Delbrück Center for Molecular Medicine in the Helmholtz Association, Berlin, Germany.*

**corresponding author (htlanger@ucdavis.edu)*

***Figure 1. Untargeted metabolomics of rat TA four weeks after constriction injury to the sciatic nerve***


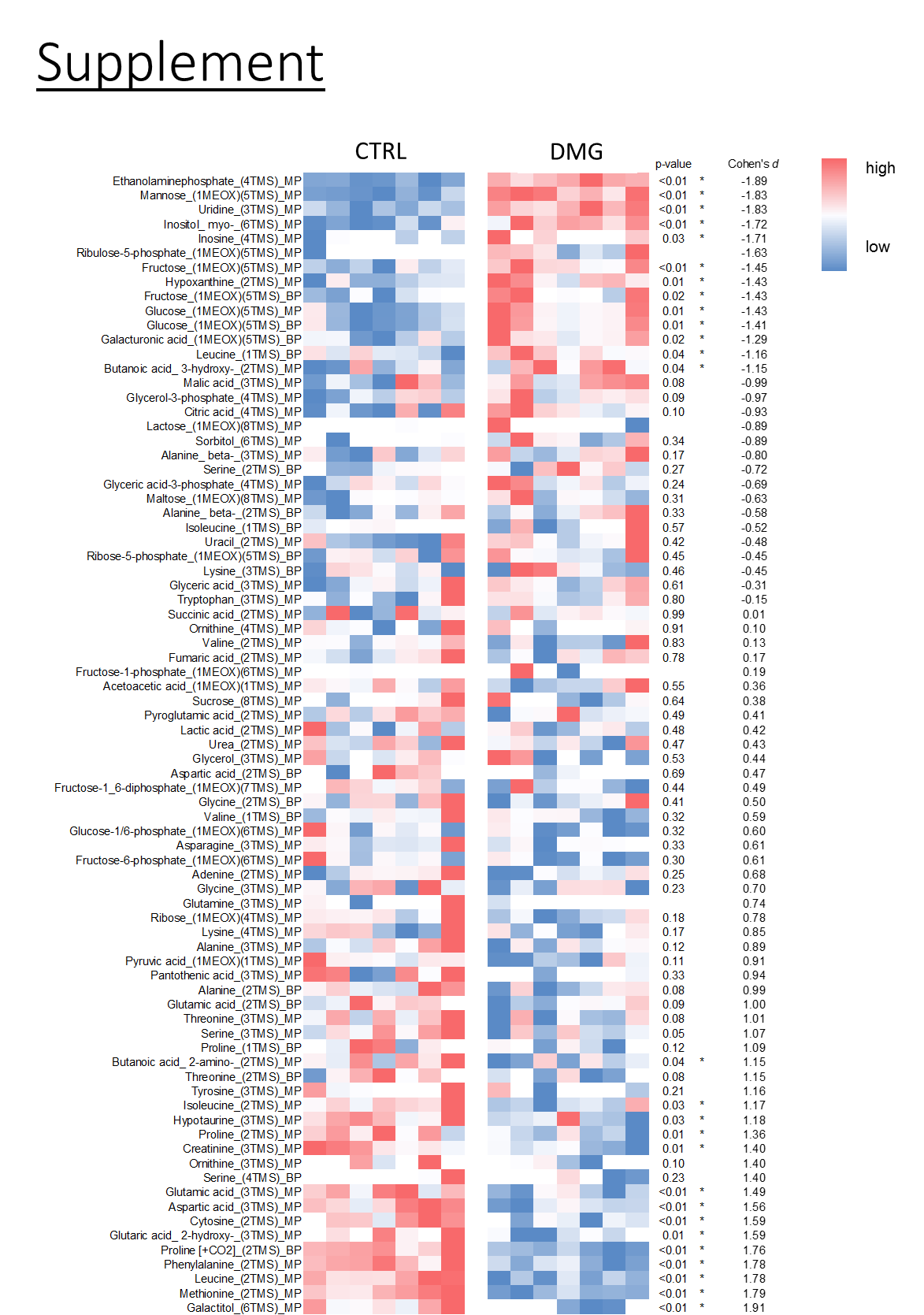


**Supplemental Figure**. Heatmap of all metabolites. After annotation, 79 polar metabolites could be identified, 27 of which were significantly altered in nerve damaged TA (DMG) compared to the contralateral control leg of the same animals (CTRL). “MP” indicates main product, “BP” indicates by product of a metabolite. Effect size (*d*) was calculated according to Cohen. Metabolites are sorted from low to high *d*.

**Original Western Blots (corresponding to Figure 3A & B as well as 4A & B in the main article) after exposure to LI-COR:**

Akt, Cyclophilin





The upper part of the membrane was probed for AKT, the lower part for Cyclophilin. Sample order is identical to the cropped images in the main figure.

pAkt, Cyclophilin





The upper part of the membrane was probed for pAKT, the lower part for Cyclophilin. Sample order is identical to the cropped images in the main figure.

AMPK, Cyclophilin





The upper part of the membrane was probed for AMPK, the lower part for Cyclophilin. Sample order is identical to the cropped images in the main figure.

pAMPK, Cyclophilin





The upper part of the membrane was probed for pAMPK, the lower part for Cyclophilin. Sample order is identical to the cropped images in the main figure.

Dysferlin, Alpha Tubulin, Cyclophilin


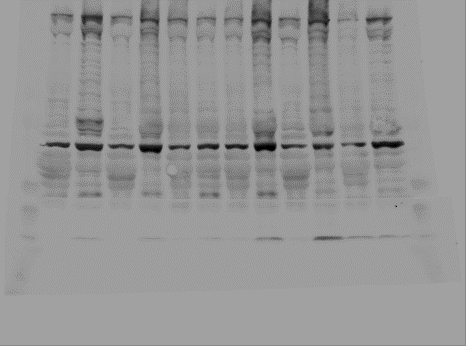


The upper part of the membrane was probed for Dysferlin and alpha Tubulin, the lower part for Cyclophilin. Sample order is identical to the cropped images in the main figure.


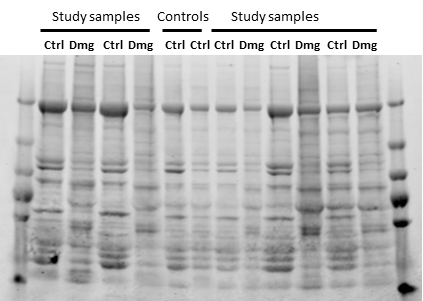


Picture of the gel at all Western Blots above (Figure 3A&, Figure 4A&B) were normalized to. The gel was stained with Coomassie Blue and imaged on a Odyssey device (LI-COR, USA).

**Western Blots added at a later stage of the manuscript (corresponding to Figure 3A & C as well as 4A & C in the main article) after exposure to BIO-RAD:**


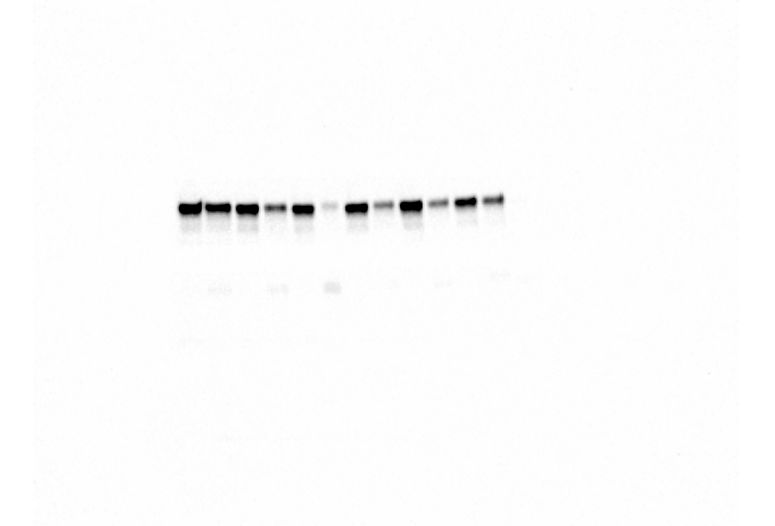


Picture of the membrane that probed for AS160, GS, GLUT4 and LC3B after auto-exposure. The pronounced bands correspond to the part of the membrane that was incubated with GS (Cell Signaling, 3893). The order of the samples alternated between healthy controls and nerve damaged TAs from left to right.


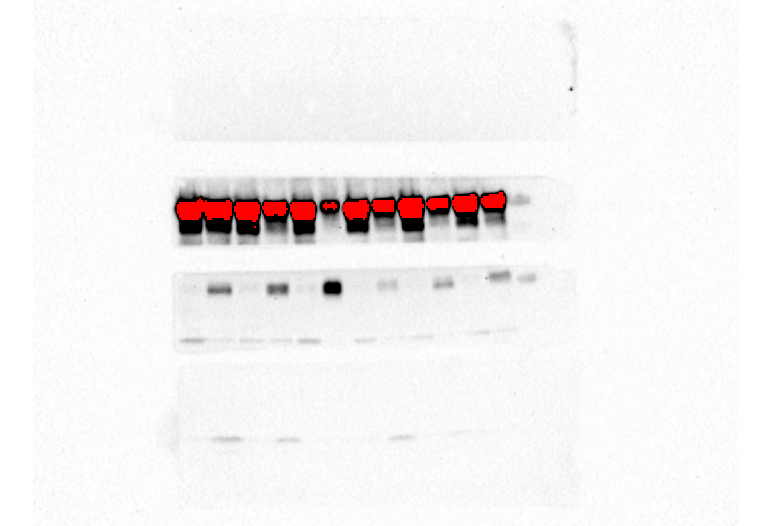


Picture of the same membrane, probing for AS160, GS, GLUT4 and LC3B after 90s of exposure. The overexposed bands belong to GS, the bands on the membrane strip below GS correspond to GLUT4. Based on the molecular weight, the lower bands on the GLUT4 strip indicate unspecific binding of the antibody. The membrane strip below GLUT4 (Santa Cruz, 53566) probed for LC3B (Cell Signaling, 2775).


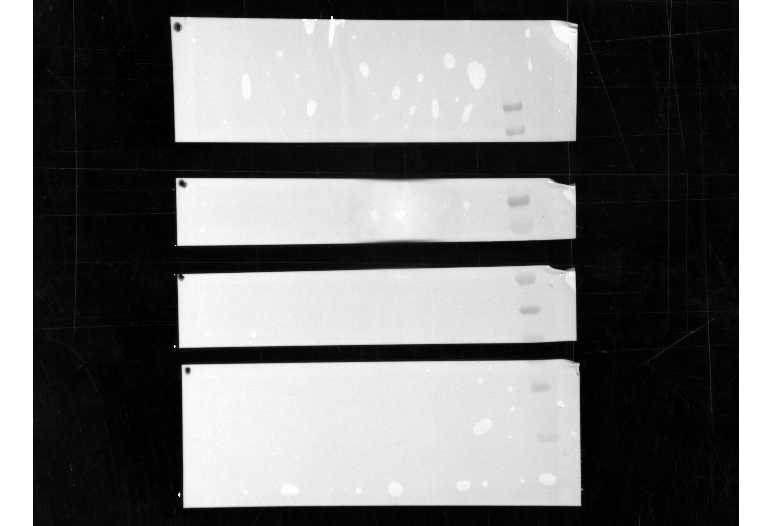


Photo of the membrane probing for AS160, GS, GLUT4 and LC3B. The ladder on the right is a PageRuler Prestained protein ladder (Thermo Scientific, USA).


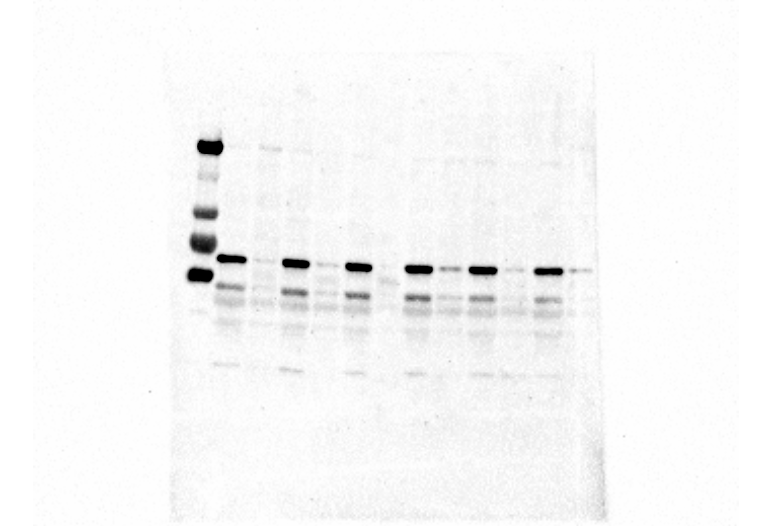


Picture of the membrane that probed for NOS (pan) (CS, 2977) after auto-exposure. The order of the samples alternated between healthy controls and nerve damaged TAs from left to right.


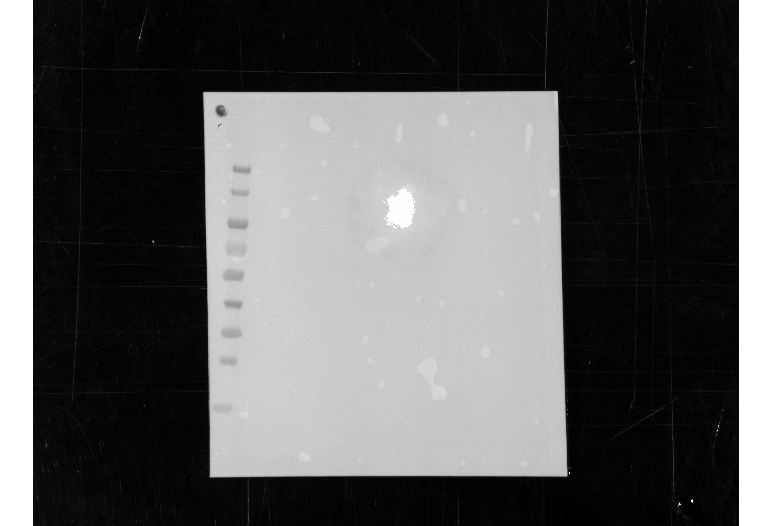


Photo of the membrane that probed for NOS (pan). The ladder on the left is a PageRuler Prestained protein ladder (Thermo Scientific, USA).


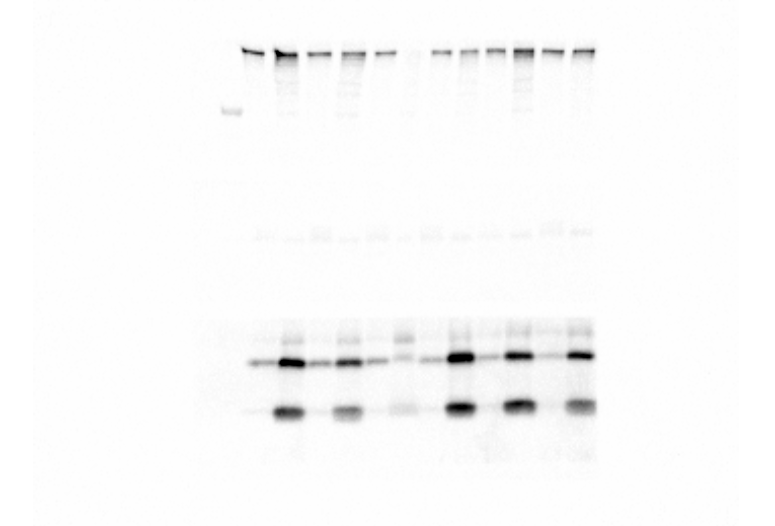


Picture of the membrane that probed for Dystrophin, GSK3 and Muscle LIM after auto-exposure. The lowest membrane strip corresponds to the part of the membrane that was incubated with muscle LIM (Santa Cruz, 166930). The order of the samples alternated between healthy controls and nerve damaged TAs from left to right.


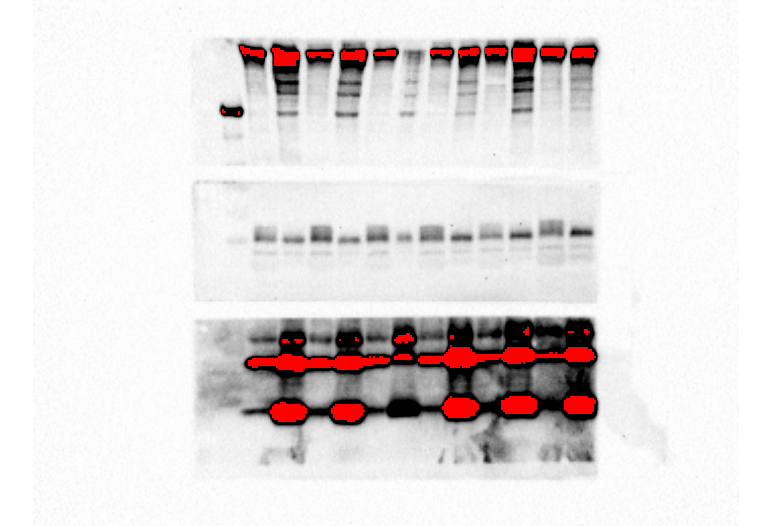


Picture of the membrane that probed for Dystrophin, GSK3 and Muscle LIM after 60s of exposure. The membrane strip in the middle corresponds to the part of the membrane that was incubated with GSK3 α/β (Cell Signaling, 9331).


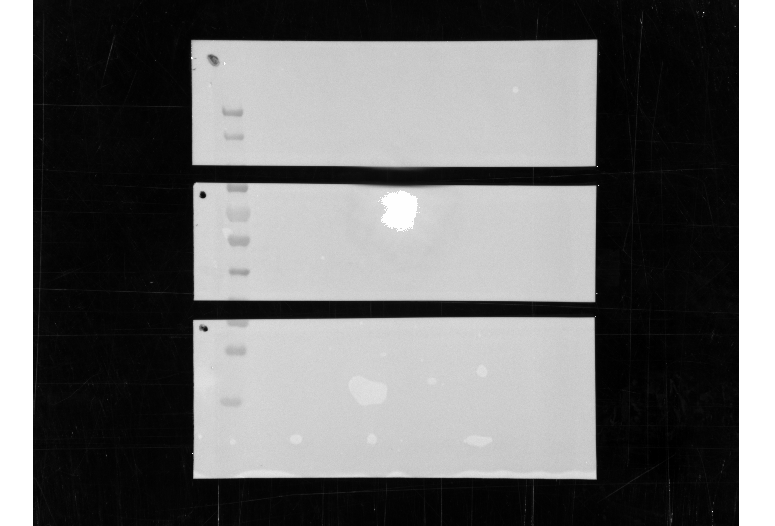


Photo of the membrane that probed for Dystrophin, GSK3 and Muscle LIM. The ladder on the left is a PageRuler Prestained protein ladder (Thermo Scientific, USA).


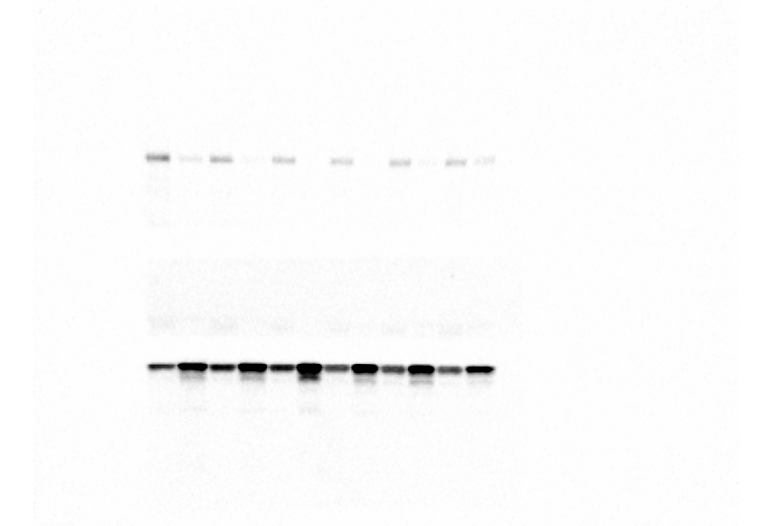


Picture of the membrane that probed for IRS1, GLUT1 and AnnexinA2 after auto-exposure. The membrane strip on the top corresponds to the part of the membrane that was incubated with IRS1 (Cell Signaling, 2382). The membrane strip on the bottom corresponds to the part of the membrane that was incubated with AnnexinA2 (Cell Signaling, 8235). The order of the samples alternated between healthy controls and nerve damaged TAs from left to right.


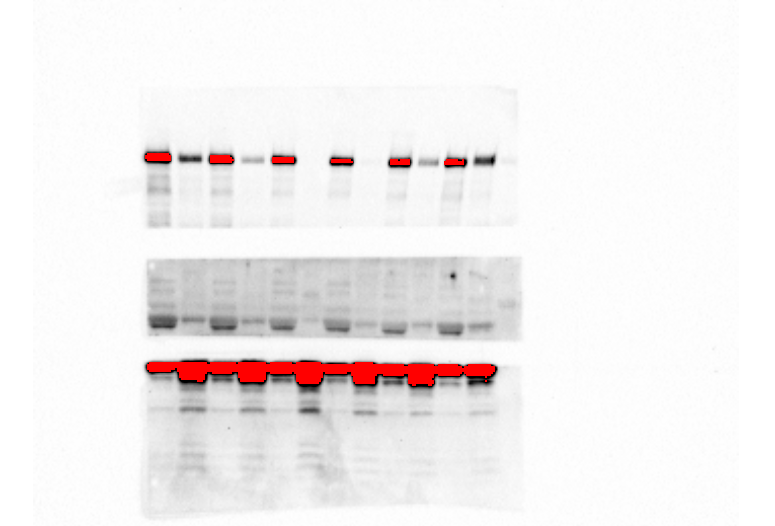


Picture of the membrane that probed for IRS1, GLUT1 and AnnexinA2 after 60s exposure. The membrane strip in the middle corresponds to the part of the membrane that was incubated with GLUT1 (Santa Cruz, 7903).


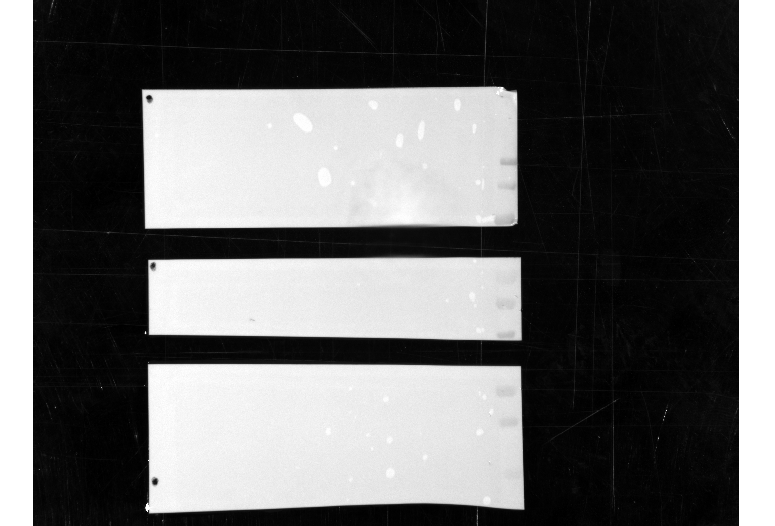


Photo of the membrane that probed for IRS1, GLUT1 and AnnexinA2. The ladder on the right is a PageRuler Prestained protein ladder (Thermo Scientific, USA).


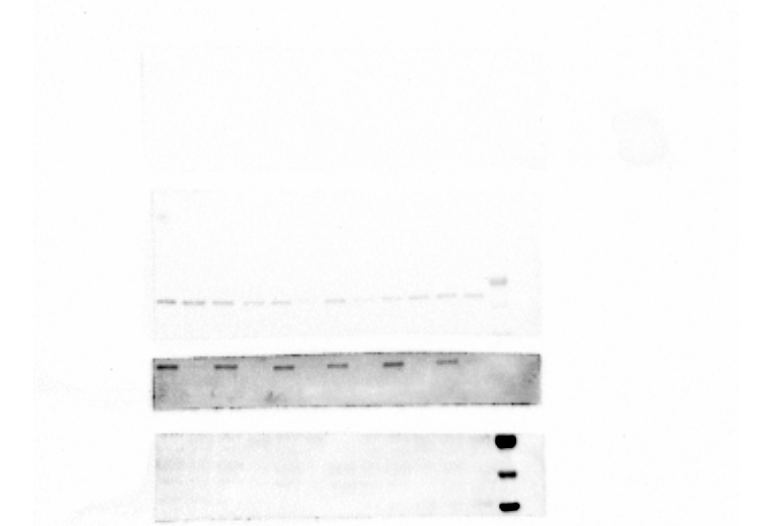


Picture of the membrane that probed for NCAM, eNOS / NOS III, PFK1 and CAMKII after auto-exposure. The membrane strip on the top corresponds to the part of the membrane that was originally originally probed (unsuccesfully) with AS160. The second membrane strip from the top corresponds to the part of the membrane that was incubated with eNOS / NOS III (BD Biosciences, 610296). The third strip from the top corresponds to the part of the membrane that was incubated with PFK1 (Santa Cruz, 31712). The strip at the bottom corresponds to the part of the membrane that was incubated with PFK1 (Santa Cruz, 31712). The strip at the bottom corresponds to the part of the membrane that was incubated with CaMK II. The order of the samples alternated between healthy controls and nerve damaged TAs from left to right.


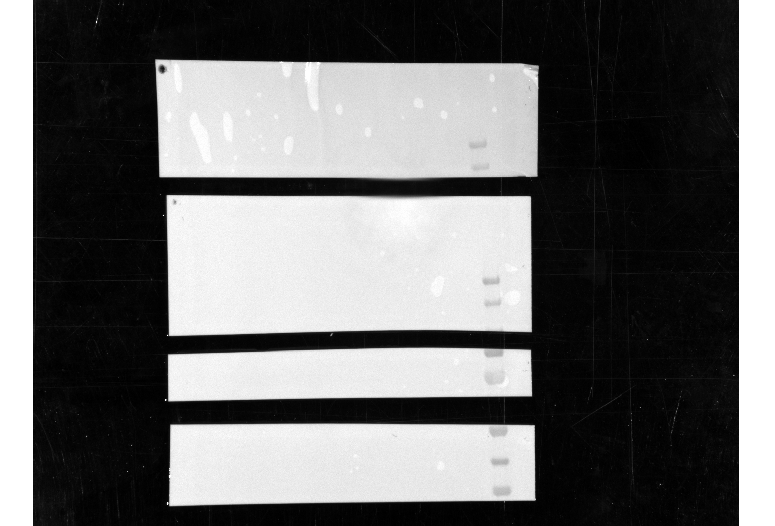


Photo of the membrane that probed for eNOS / NOS III and PFK1. The ladder on the right is a PageRuler Prestained protein ladder (Thermo Scientific, USA).


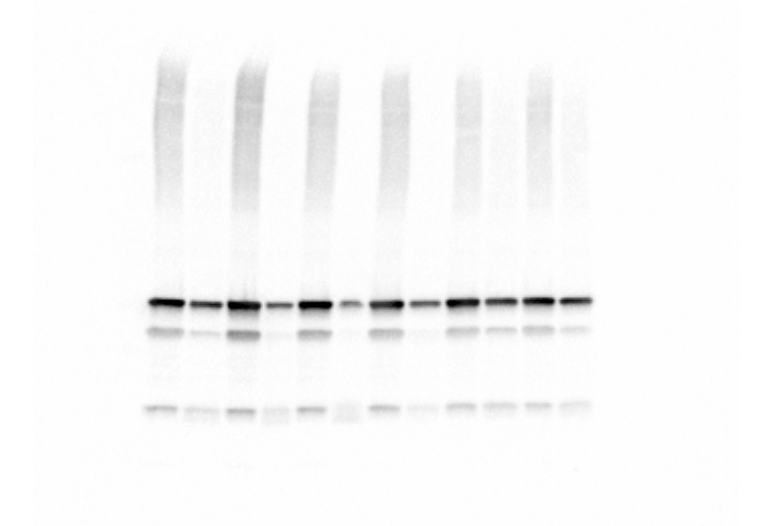


Picture of the membrane that probed for total OXPHOS (Abcam, MS604-300) after auto-exposure. The order of the samples alternated between healthy controls and nerve damaged TAs from left to right.


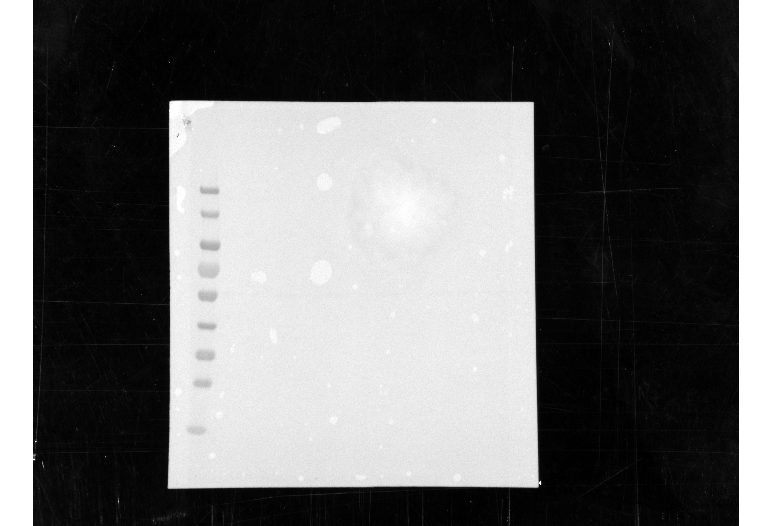


Photo of the membrane that probed for total OXPHOS. The ladder on the left is a PageRuler Prestained protein ladder (Thermo Scientific, USA).


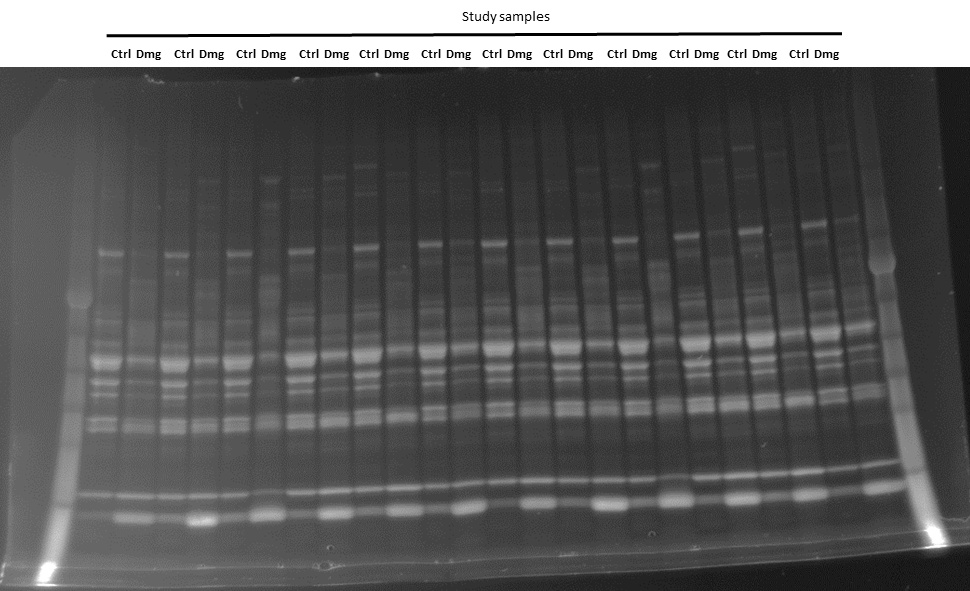


One of the gels the Western Blots during revisions of the manuscript were normalized to. The gel is a Criterion TGX Stain-Free gel (BIO-RAD, USA) and indicates total protein content of each lane after being exposed to UV light for approximately one minute.
